# Lived experiences of psychiatric patients with mood disorders who attended group therapy facilitated by professional psychiatric nurses

**DOI:** 10.4102/curationis.v43i1.2114

**Published:** 2020-06-11

**Authors:** Hester M.P. Visagie, Marie Poggenpoel, Chris Myburgh

**Affiliations:** 1 Department of Nursing, University of Johannesburg, Johannesburg, South Africa; 2 Department of Educational Psychology, University of Johannesburg, Johannesburg, South Africa

**Keywords:** group therapy, lived experiences, psychiatric nurses, stabilised acute psychiatric patients, acute inpatient unit

## Abstract

**Background:**

According to the World Health Organization (WHO), up to 25% of people worldwide will develop mental health disorders during their lifetime. Patients admitted to acute inpatient units for mood disorders experience emotional distress. Group therapy has the potential to foster the therapeutic change through specific therapeutic mechanisms. Psychiatric nurses working in inpatient units are in a unique position to offer group therapy.

**Objectives:**

Explore and describe stabilised acute psychiatric patients with mood disorders’ lived experiences of group therapy facilitated by psychiatric nurses. Make specific recommendations for psychiatric nurses to facilitate constructive group therapy for stabilised acute psychiatric patients with mood disorders in an inpatient unit.

**Method:**

A qualitative, exploratory, descriptive and contextual design was used in the study. A purposive sample of all patients with mood disorders older than 18 years admitted to inpatient units who participated in group therapy was made. Data were collected through conducting phenomenological interviews, observation and field notes. Interviews focussed on the following open question: ‘How did you experience group therapy facilitated by the psychiatric nurses?’ An independent coder analysed the data by using thematic coding. Measures to ensure trustworthiness were applied. The following four ethical principles were adhered to: autonomy, non-maleficence, beneficence and justice.

**Results:**

Three themes emerged from this study. Theme 1 entailed the psychological experiences of patients attending group therapy. Theme 2 highlighted the interpersonal experiences of patients. Theme 3 evolved around patients’ experiences outside group therapy. Patients initially experienced attending group therapy as anxiety provoking. However, negative psychological experiences soon transformed into positive psychological experiences.

**Conclusion:**

The findings of this study were used to make specific recommendations to facilitate constructive group therapy for patients with mood disorders.

## Introduction and background

In this study, the focus is on the lived experiences of acute psychiatric patients with mood disorders who attended group therapy facilitated by professional psychiatric nurses. Mental illness poses a challenge worldwide and represents an important source of disability and the global disease burden in both first and third world countries. According to the Department of Health (2013:5), mental, physical and social health is one of the vital strands of human life, which have a direct impact on the persons’ social world (Sadock, Sadock & Ruiz 2015:1400). This is especially important when considering that the World Health Organization (WHO) estimates that up to 25% of people worldwide will develp mental health disorders during their lifetime (WHO 2013:32). Amongst mental disorders, mood disorders, in particular, and depression are described as having the highest prevalence rate.

Mood disorders are characterised by disturbances in affect, mood, cognitive functioning and are often accompanied by marked deterioration in occupational and social functioning (Sadock et al. 2015:347). Patients with mood disorders often experience hopelessness and feelings of worthlessness (Uys & Middleton 2014:359). These patients feel overwhelmed and can no longer fulfil daily activities of living (Smith 2012:51; Stuart 2013:293). Mood disorders also lead to diminished capacity to fulfil role expectations in the community, which have an adverse impact on interpersonal relationships (Cristia et al. 2013:584).

On the basis of empirical studies (Bernhardsdottir, Champion & Skärsäter 2014:680, Chetty & Hoque 2013:30; Lorentzen & Ruud 2014:220), psychotherapeutic interventions, inclusive of individual, family and group therapy, are considered effective in the treatment of mood disorders. Group therapy, in particular, has been described as a powerful treatment intervention in psychological disorders (Singh 2014:154) and focusses on specific mechanisms involved in the therapeutic change (Caruso et al. 2013:n.p.).

Globally, psychiatric nurses play an important role in the multidisciplinary team in inpatient units for acute psychiatric patients with mood disorders. These psychiatric nurses are in a unique position to offer group therapy to patients experiencing emotional turmoil. Although the current nursing care programme in the unit where the research was conducted includes cognitive behaviour group therapy sessions facilitated by psychiatric nurses, the lived experiences of stabilised acute psychiatric patients with mood disorders who attended group therapy facilitated by professional psychiatric nurses have not been explored, nor described.

The identified knowledge gap led to the following research question: How do stabilised acute psychiatric patients with mood disorders experience group therapy facilitated by psychiatric nurses?

## Purpose of the study

Therefore, the purpose of this study was to understand the lived experiences of stabilised acute psychiatric patients with mood disorders who attended group therapy facilitated by professional psychiatric nurses.

## Definition of key concepts

### Group therapy

Group therapy is defined as a (Uys & Middleton 2014):

[*S*]tructured/semi-structured process of therapeutic intervention in which behavioural and emotional responses of individual members of a group towards one-another and towards the group leader are utilized to improve mental health and combat mental illness. (p. 241)

Group therapy therefore provides a safe learning environment for the facilitation of education and change pertaining to emotional experiences and behavioural responses. Group therapy is a learning and problem-solving process. This process involves both the individuals in the group, the self as well as the collective group members – the collective self.

### Lived experiences

Exploration of human experiences is rooted in the theory of phenomenological approaches. Humans are seen as self-interpreting with the unique quality to perceive, experience and assign meaning to their life experiences (Gray Grove & Sutherland 2017:66). During this study, the researcher explored and described the lived experiences of stabilised acute psychiatric patients with mood disorders, attending group therapy facilitated by psychiatric nurses.

### Psychiatric nurses

Psychiatric nurses play an essential role in the treatment of patients with mood disorders. In South Africa, the South African Nursing Council – as the regulatory body of the nursing profession – provides practice guidelines, which state that professional nurses with an additional and specialised mental health care nursing qualification should offer high-quality and evidence-based patient care (South African Nursing Council 2016). In this study, psychiatric nurses refer to professional nurses in the unit qualified in psychiatric nursing care.

### Psychiatric nursing

The International Council for Nurses (2014) defines nursing practice as the:

[*A*]utonomous and collaborative care of individuals of all ages, families, groups and communities, ill or well in all settings. It includes health promotion, prevention of illness, and the care of the ill, disabled and dying people. (n.p.)

Psychiatric nursing is a speciality field in the nursing profession where the therapeutic relationship between psychiatric nurses and patients is seen at the core of psychiatric nursing practice. Psychiatric nursing interventions aim to prevent mental illness and promote mental health (Uys & Middleton 2014:185, 833). In this study, psychiatric nursing refers to the nursing care of patients with mood disorders based on nursing assessment, formulation of a nursing diagnoses, planning and implementation of care and the evaluation there-off with the aim to strengthen functional life skills and modify dysfunctional life skills (Uys & Middleton 2014:189, 195).

### Stabilised acute psychiatric patients

Psychiatric patients with acute mental illnesses are characterised by significant and distressing symptoms of mental illness requiring immediate treatment (Government of Malta 2017:n.p.). For these psychiatric patients to be psychiatrically stable, the acute mental illness must be treated (Bitterman 2018:n.p.). In this study, stabilised acute psychiatric patients refer to individuals with mood disorders who have been treated and do not experience any mood or related disturbance.

### Acute inpatient unit

Acute psychiatric inpatient units are described as admission units in psychiatric hospitals where acutely ill patients are admitted with the aim to provide short-term mental health care, treatment and rehabilitation (Sobekwa & Arunachallam 2015:2).

## Research design and method

A qualitative, exploratory, descriptive, contextual design was used in this study based on a phenomenological approach (Creswell & Poth 2018:29; Gray et al. 2017:29, 66). A phenomenological approach based on a humanistic paradigm aims to explore and describe the lived experiences, perceptions and behaviour of a person or group of people, therefore making sense of reality as experienced by the individual or group when little is known about the subject (Gray et al. 2017:62; Moon et al. 2016:n.p.). Therefore, a phenomenological descriptive approach (Holloway & Galvin 2017:219, 227) provided the cornerstones for this study as it explored and described the lived experiences of acute psychiatric patients who attended group therapy facilitated by professional psychiatric nurses.

### Population and sampling

The target population consisted of all acute stabilised psychiatric patients with mood disorders admitted to a psychiatric unit who participated in group therapy facilitated by psychiatric nurses. A purposive sampling method was used (Creswell 2016:109; De Vos et al. 2011:232; Fain 2017:180; Holloway & Galvin 2017:144). The criteria for inclusion were all patients admitted to the unit with age above 18 years. Participants could be male or female and had to have an adequate grasp of English. Participants were interviewed upon discharge. Participants were requested to sign consent for the interview, as well as for the audio recording of the interview.

### Data collection

Data were collected by means of conducting individual in-depth phenomenological interviews, observation and field notes made by the researchers during the interviews. Interviews focussed on the following open broad question: How did you experience group therapy by the psychiatric nurses? Eight participants were interviewed.

### Data analysis

In qualitative descriptive studies, the purpose of data analysis is to identify important characteristics, themes and patterns inherent to a specific phenomenon of interest to the researcher (Fitzpatrick 2018:155; Sohn et al. 2017:139). In this study, data were analysed according to Creswell’s principles of data analysis (Creswell 2016:154). Recorded interviews and the transcriptions thereof were utilised to organise raw data. Thematic coding was applied to categorise raw data into smaller parts to identify important themes and categories. Field notes and observations made during the interviews were incorporated. An independent coder experienced in qualitative research was utilised to analyse the data and a consensus meeting was held.

### Trustworthiness

The trustworthiness of a study refers to the extent to which the researcher can provide and ensure transferability, provide true findings and interpret the findings of the study into meaningful units (Gray et al. 2017:450). In this study, the researchers applied Guba’s model of trustworthiness (Denzin & Lincoln 2011:92; Yüksel & Yildirim 2015:13). The following four criteria of trustworthiness were utilised: transferability, credibility, dependability and confirmability. Triangulation was ensured through interviews, observation and field notes.

### Findings

A description of the demographics of the study participants and themes identified during the exploration of the lived experience of patients with mood disorders who attended group therapy facilitated by psychiatric nurses is as follows.

### Description of the demographics of the participants

The participants who were interviewed consisted of four men and four women. The male participants were 18, 32, 44 and 53 years of age. The female participants were 20, 24, 22 and 40 years of age. Thus, the participants’ age profile ranged from 18 to 53. Table 1 presents the demographic data of the eight in-depth phenomenological interviews conducted with the participants.

**TABLE 1:** Demographic data of patients with mood disorders who attended group therapy by psychiatric nurses.

Interviews	Gender	Age	Ethnic relationship	Home language	Marital status	Employment status	Level of education
**Participant 1**	Male	44	Mixed descent	Sotho	Married	Employed	Tertiary (university degree)
**Participant 2**	Female	24	African	Sotho	Single	Employed	Grade 12
**Participant 3**	Male	18	White	Afrikaans	Single	Unemployed	Grade 12
**Participant 4**	Female	20	Mixed descent	Afrikaans	Single	Student	Tertiary (university degree)
**Participant 5**	Male	32	African	Sotho	Single	Unemployed	Grade 10
**Participant 6**	Female	40	African	Sotho	Married	Unemployed	Grade 12
**Participant 7**	Female	32	African	Sotho	Married	Unemployed	Grade 11
**Participant 8**	Male	53	White	Afrikaans	Married	Unemployed	Tertiary (diploma)

*Source*: Visagie, H.M.P., 2019, ‘Patients with mood disorders’ lived experiences of group therapy by psychiatric nurses’, MSc Psychiatric Nursing minor dissertation, University of Johannesburg, Johannesburg, p. 44

### Findings of the interviews: Themes of experiences of psychiatric patients with mood disorders who attended group therapy facilitated by psychiatric nurses

Three themes were identified from the data obtained from this study. Table 2 presents the findings of the interviews.

**TABLE 2:** Patients with mood disorders lived experiences of group therapy by psychiatric nurses.

Themes	Description
Theme 1	Patients’ psychological experiences of group therapy facilitated by psychiatric nurses
Theme 2	Patients’ interpersonal experiences of group therapy facilitated by psychiatric nurses
Theme 3	Patients’ experiences outside group therapy

*Source*: Visagie, H.M.P., 2019, ‘Patients with mood disorders’ lived experiences of group therapy by psychiatric nurses’, MSc Psychiatric Nursing minor dissertation, University of Johannesburg, Johannesburg, p. 52

#### Theme 1: Patients’ psychological experiences of group therapy facilitated by psychiatric nurses

Patients with mood disorders admitted to inpatient units for mood disorders experience severe emotional turmoil. These patients often feel overwhelmed, incapacitated, helpless and hopeless, unable to fulfil daily activities of daily living (Visagie 2019:45). They experienced a wide range of psychological emotions. Their experiences ranged from negative to positive.

During the initial phase of group therapy, participants had negative psychological experiences resulting in emotional distress. Entering the group setting for the first time was anxiety provoking. Participants were reluctant to express their feelings in the group setting and felt afraid of being judged and labelled.

Fear of being judged and labelled was supported by the following quotes:

‘Yo, at first I, I didn’t want to open up. I was feeling like, they are going to think I’m weak.’ (P2, female, 24 years, Grade 12 educational level)

‘I was a bit nervous, because I’m not really a group person. I felt nervous and anxious.’ (P3, male, 18 years, Grade 12 educational level)

As the group process progressed, participants’ negative psychological experiences transformed into positive psychological experiences. Group members started to experience the group setting as a safe space and felt less awkward and vulnerable when sharing intimate feelings. As group cohesion developed, group members experienced feeling safe and expressed not feeling alone anymore. Group cohesion allowed members to experience a sense of belonging. Group members feel valued, respected and cared for (Uys & Middleton 2014:243). Group members developed a sense of belonging and sharing as stated in the quotations below:

‘But then as time went by, I realised that keeping quiet won’t help me. I have to speak up; I have to talk so that I can heal, and at the end of the day I felt loved and accepted.’ (P2, female, 24 years, Grade 12 educational level)

‘I felt good. I felt appreciated. I felt like a human being again. I felt that I could voice my opinion in a constructive manner. I felt like I meant something.’ (P1, male, 44 years, tertiary educational level)

Attending group therapy facilitated by psychiatric nurses enabled participants to experience feeling loved and accepted. Members felt free to explore and verbalise their most intimate feelings. Group members realised that they were accepted and valued irrespective of what they disclosed during group sessions. Participants stated the following in terms of expressing intimate personal feelings:

‘In some households you’re not allowed to express your emotions or otherwise there is trouble. It is regarded as talking back or being arrogant or something like that.’ (P3, male, 18 years, Grade 12 educational level)

‘Yes, they were not judgemental at all. They were like listening rather than saying, they were a kind ear as well.’ (P4, female, 20 years, tertiary educational level)

Group members were of the opinion that they could trust the psychiatric nurses facilitating the groups as well as the other group members. They perceived the group setting and process as authentic. The following quote supports this statement:

‘The point is, everything being said in the group, stayed in the group and what they said was actually what happened. Nobody gossiped.’ (P3, male, 18 years, Grade 12 educational level)

Participation in group therapy provided a therapeutic environment fostering self-reflection. Participants could reflect on their own thoughts, feelings and related behavioural responses by observing and analysing thought processes and behavioural responses of other group members. Participants reflected the following pertaining to self-reflection:

‘Maybe being alone, that is what caused me to think those negative thoughts. If I think a negative thought and I tell you, you are going to help me to get out of that negativity.’ (P2, female, 24 years, Grade 12 educational level)

‘People are not the same; I always thought that people were cruel and rude. I learned some people are loving, some people are kind, some people will care about you even though they don’t even know you.’ (P7, female, 32 years, Grade 11 educational level)

Exposure to therapeutic mechanisms underpinning group therapy facilitated by psychiatric nurses, and being able to reflect on one’s own feelings, perceptions, thought processes and related behavioural responses, provided participants with a foundation to take responsibility for their own lives, feelings, perceptions and behaviour. Group therapy thus fostered a personal change. This statement is supported by the following quotes:

‘Change won’t come by itself, that you play a certain role.’ (P4, female, 20 years, tertiary educational level)

‘I was feeling stupid because every time something happens the negative thought or negative emotion was the first to come. I wouldn’t just calm down and take time to look at the situation.’ (P2, female, 24 years, Grade 12 educational level)

Attending group therapy provided participants with the opportunity to interact with the psychiatric nurses as facilitators, as well as other group members. Through their interaction with others during group therapy sessions, participants became aware that they had the potential to make valuable contributions to the group process as well as to the experiences of other group members. This enabled group members to experience self-worth. Experiencing self-worth was expressed in the following quotes:

‘I feel like I have a potential, a lot of potential. I was just looking down on myself. I’ve a lot of potential.’ (P2, female, 24 years, Grade 12 educational level)

‘It was as if I worked on a skill that I actually did not know I needed to work on. That is a good thing, it’s always a good thing to receive direction, growing, learning new things or acquiring like a new skill.’ (P4, female, 20 years, tertiary educational level)

Patients with emotional distress often experience being alone and a belief that nobody understands their emotional turmoil. These patients isolate themselves from others and experience being alienated from their previous world of interaction. Participation in group therapy enabled patients to become aware that others are also struggling and that they are not alone. One participant expressed the following regarding not feeling alone anymore.

‘By sharing with others, hearing their stories helped me realise that I’m not alone, you know.’ (P2, female, 24 years, Grade 12 educational level)

According to Yalom and Leszcz (2005:6), patients enter group therapy with the pre-conception that they are alone in their emotional struggle. Group therapy provides patients with the opportunity to realise that they are not alone and that other people also experience emotional distress. Knowing that others go through emotional turmoil, as well as feeling understood, provides patients with emotional relief.

#### Theme 2: Patients’ interpersonal experiences of group therapy facilitated by psychiatric nurses

When patients’ interpersonal experiences of group therapy facilitated by psychiatric nurses were explored, it became evident that participants distinguished between their interaction with the psychiatric nurses and their interaction with other members of the groups. Although participants experienced both negative and positive interpersonal experiences, the majority of participants reflected positive interpersonal experiences.

The nurse–patient relationship seemed to be a very prominent component in the group therapy sessions. Group therapy sessions were conducted by different psychiatric nurses in the unit, and participants at times experienced a lack of compassion. The following quote highlights one participant’s perceived lack of compassion:

‘Yo, some of them were rude sometimes when asking them for help or telling them I’m not feeling well. I was afraid of telling them, but some of them. I felt like she’s my mother.’ (P2, female, 24 years, Grade 12 educational level)

However, the majority of the participants reflected experiencing compassion, hope, feeling safe and being cared for in their interaction with the psychiatric nurses. Patients’ personal change and empowerment are vested in the therapeutic nurse–patient relationship. Group development can evolve effectively only if group members experience feeling safe, respected and valued. Instilling hope was described as an important therapeutic factor in the therapeutic group process for patients with mood disorders. The following quotes support this statement:

‘And I really felt very close to them, they cared about me. When I said I have pain, they gave me something and were nice to me.’ (P6, female, 40 years, Grade 12 educational level)

‘Here I feel like I’m supported. I get the support I’ve been longing for, you know. I’ve got it and they were pushing me, showing they were willing to help you, we are here.’ (P2, female, 24 years, Grade 12 educational level)

Feeling physically and emotionally safe, valued and respected is a core necessity in human existence and self-actualisation. Contributing in a positive manner to society, other people and life itself was also highlighted during this study. The following quote emphasised this aspect:

‘I’ve learned that everyone has a heavy load to carry and we forget to just take a moment to listen to others and observe others and experience others. Other people are also going through things. Now, you see it from a different angle and realise maybe it is not that bad. People have also come into bad situations and they are getting through that situation, they are working.’ (P4, female, 20 years, tertiary educational level)

No person lives entirely in solitude. Being part of a group is at the very core of human existence. Humans depend on each other for survival (Visagie 2019:67). All human beings learn to interact with others since the very beginning of life. The importance of being supported by others became evident when patients’ interpersonal experiences of interaction with other group members were explored. The following quote highlights the importance of feeling supported by others:

‘It was helping me and I felt like I’m loved. There’s somebody out there who loves me. I come from a family where I’m not supported and here I feel like I’m supported.’ (P2, female, 24 years, Grade 12 educational level)

Acknowledging intimate vulnerable feelings can be quite challenging for most people. The therapeutic space of the group setting provided a safe space to express feelings and to realise that it is okay to share feelings. Group therapy provided participants with ‘a close-up’ insight into the emotional component of human existence; they expressed their own feelings, and realised that other group members also experienced the same or different emotions. Participants reflected the following pertaining to expressing feelings in the group setting:

‘I felt when I told my problems, I felt better, my mind felt better, because when I stay with my problems my emotions will be high. So that’s why, I realised when I’m in the group I must tell my problems, like they did it. So I learned that from the other patients. I learned from their experience how to solve my problems.’ (P5, male, 32 years, Grade 10 educational level)

‘They will benefit because like I said other people are experiencing what you are experiencing too and it is okay to express your emotions. It’s okay, it is a safe place.’ (P4, female, 20 years, tertiary educational level)

Humans have a deep-seated need to experience self-actualisation. Self-actualisation is vested in giving meaning to one’s own life as well as to contribute to the life experiences of others. Furthermore, personal achievement, enjoyment and interaction with others seem to contribute to meaningful life experiences. Participants reflected the following pertaining to spending time with other people:

‘I have learned to show my emotions, I could acknowledge my emotions; show emotions to others I am in a relationship with and other aspects. I’ve learned that a person does not live alone.’ (P1, male, 44 years, tertiary educational level)

‘I was that person who was always alone, isolating myself from other people, but then I’ve learned that it’s gonna help me. I will enjoy life, spending time with people. Maybe just being alone, that is what was causing me to think those negative thoughts.’ (P2, female, 24 years, Grade 12 educational level)

The group setting provided a therapeutic space where participants could feel safe and accepted by others. Participants were of the opinion that they could share their most intimate feelings without being judged. Participants reflected the following when expressing their experiences of the therapeutic environment within the group setting:

‘In my family, we were taught that crying was a sign of weakness, and here it was okay to cry, it was accepted. I felt like this is a place, I don’t want to say home, but it was a safety net.’ (P4, female, 20 years, tertiary educational level)

‘First of all, greet, create a welcoming environment for the patients. Make sure they understand and relax and make them feel free to talk. I didn’t want to open up, but eventually I opened up, because some guy made me understand opening up is going to help me and it did help me. Make us understand why we are in the session and how it’s going to help us.’ (P2, female, 24 years, Grade 12 educational level)

Attending group therapy provides a safe space for self-disclosure, self-awareness and expressing intimate feelings. The therapeutic group process provides an opportunity for corrective learning when patients struggle with expressing emotions (Uys & Middleton 2014:243; Yalom & Leszcz 2005:89).

#### Theme 3: Patients’ experiences outside group therapy

Shives (2008:153) defines the therapeutic environment as inclusive of the physical environment, patients and all the staff involved in rendering the service. The overarching aim of a therapeutic environment is to empower patients to utilise problem-solving skills to cope with intrapersonal challenges, interpersonal challenges, trauma, losses and environmental stressors.

Patients’ experiences of group therapy evolved around the perceived therapeutic environment and interaction with psychiatric nurses and other group members outside the group setting. Psychiatric nurses and group members develop a perception of each other as human beings; thereafter, the perception of the psychiatric nurse or the patient follows and relates to perceptions within the group setting extending to perceptions of one another outside the group setting. The therapeutic environment should foster therapeutic relationships between psychiatric nurses and patients, and between patients themselves (Uys & Middleton 2014:285).

During this study, it became evident that participants valued both the therapeutic space within the group setting as well as the therapeutic space within the ward. Patients experienced the therapeutic environment outside the group setting as a safety net promoting interpersonal learning and building trust. Participants reflected the following pertaining to experience acceptance outside the group setting:

‘The professional nurses were really very good, sometimes they came in the morning and asked how you slept; or they would ask about my pregnancy and if the baby was moving.’ (P7, female, 32 years, Grade 11 educational level)

‘They listened and sometimes we played games all together. We go to OT (occupational therapy), we laugh and we smile at each other. When I needed something they attended to my needs, also out of the groups.’ (P6, female, 40 years, Grade 12 educational level)

The findings of this study revealed that the interaction between the participants within the group setting was closely related to the interaction between the participants outside the group setting. Therapeutic interaction outside the group setting fostered a sense of safety, being valued and cared for. The following quotes support this statement:

‘It wasn’t in the group session; it was outside the group session. One of the group members and the sister came with like a bundle of tissues when I started crying, and she did not really say anything, she just rubbed my back, like comforting.*’* (P4, female, 20 years, tertiary educational level)

‘Yaa, and sometimes when we ate and they talked to me and said nice things, so they cared about me.’ (P6, female, 40 years, Grade 12 educational level)

Patients with mood disorders admitted to acute inpatient units feel vulnerable and often do not know what to expect. Fear of the unknown hampers feeling secure and safe on an emotional and physical level. During group therapy sessions, members learned about the demographic particulars of fellow members. The group space provided a window to other participants’ intimate emotional experiences. Sharing intimate emotional experiences resulted in participants feeling safe.

According to the literature, the therapeutic environment provides a safety net that should promote interpersonal learning, autonomy, self-esteem, trust, self-reflection, self-reliance and the ability to relate to others with the aim to return to the community as a functional member of society (Shives 2008:153; Uys & Middleton 2014:285).

### Ethical considerations

Permission to conduct the study was obtained from the Free State Department of Health and the Chief Executive Officer of the institution where the study was conducted. Ethical clearance was obtained from the Faculty of Health Sciences Research Ethics Committee (# REC-01-35-2018) and National Health Research Review Board (University of Johannesburg) (HDC – 01-48-2018). The researcher obtained written informed consent from the study participants to participate in the study as well as written informed consent to audio record the interviews. During this study, the researcher herself was not involved in conducting group therapy and was not present in any group therapy sessions. The researcher applied the following four ethical principles according to (Dhai [AQ1]& McQuoid-Mason 2011:69) autonomy, non-maleficence, beneficence and justice.

## Discussion of the findings

A discussion of the findings follows below as conceptualised in the existing literature sources.

### Participants’ experiences of group therapy facilitated by psychiatric nurses ranged from negative to positive psychological experiences

Patients suffering from mood disorders experience intense emotional turmoil. These patients feel vulnerable and are affected by cognitive and physical impairment (Kneisl & Trigoboff 2014:5; Uys & Middleton 2014:195, 358). According to the WHO, the overall well-being of individuals, families and society entails the following (WHO 2013):

Mental health is defined as a state of wellbeing in which every individual realizes his or her own potential, can cope with the normal stresses of life, can work productively and fruitfully and is able to make a contribution to the community. (p. 6)

Psychotherapeutic interventions, inclusive of individual, family and group therapy, are considered effective in the treatment of mood disorders (Bernhardsdottir et al. 2014:680; Chetty & Hoque 2013:30; Lorentzen & Ruud 2014:220). Group therapy specifically has been described as a powerful treatment intervention in mood disorders with the aim to not only induce symptom relief, but also to enhance the therapeutic growth and change (Caruso et al. 2013:n.p.; Singh 2014:154).

Joining the unfamiliar setting of group therapy sessions facilitated by psychiatric nurses for the first time led to increased levels of anxiety and discomfort. Group members feared being judged and labelled. To be able to facilitate constructive group therapy for patients with mood disorders, psychiatric nurses should be aware of these vulnerabilities. Awareness of patients’ vulnerabilities emphasises the importance of creating a safe therapeutic space in the group setting to minimise negative psychological experiences and enhance positive psychological experiences.

During the initial phase of group therapy, group members were unsure whether they could trust the psychiatric nurses facilitating the group therapy sessions and fellow group members. Trust is described as a dynamic process and forms an integral part of the therapeutic nurse–patient relationship (Rørtveit et al. 2015:196). Establishing ground rules and emphasising confidentiality ensured that group members perceived the group setting as a safe space for self-disclosure (Kneisl & Trigoboff 2014:611).

As the group therapy process developed, negative psychological experiences transformed into positive psychological experiences. Patients experiencing mood disorders often believe that no one understands what they are going through. Sharing intimate feelings in the group setting without being judged created a sense of belonging. Group members felt valued, accepted and supported. Group cohesion has been described as an important dynamic in the therapeutic group process (Uys & Middleton 2014:243).

Participating in group therapy facilitated by psychiatric nurses provided a platform where participants could gain insight into their own life experiences and reflect on their own feelings, thoughts and related behavioural responses. Observing and analysing the thought processes and behavioural responses of others facilitated this process. Self-reflection fostered personal change and enabled participants to take responsibility for their own lives, feelings, perceptions and behaviour.

Participating in group therapy facilitated by psychiatric nurses allowed participants to interact with other group members and psychiatric nurses in a meaningful manner. Participants realised that they could make meaningful contributions to the life experiences of other members. This enhanced a sense of personal and collective self-worth (Uys & Middleton 2014:243; Yalom & Leszcz 2005:27).

### Patients’ interpersonal experiences of group therapy facilitated by psychiatric nurses

This study revealed that participants experienced both negative and positive interpersonal experiences during their engagement with the psychiatric nurses facilitating the group therapy sessions. Participants emphasised and linked their experience of the therapeutic nurse–patient relationship within the group setting to their experience of the therapeutic nurse–patient relationship outside the group setting.

Different psychiatric nurses facilitated different group therapy sessions and some participants experienced some of the psychiatric nurses lacking compassion. When interpersonal challenges occurred outside the group setting, participants entered group therapy sessions with pre-conceived perceptions of not being valued and cared for. Participants then perceived the psychiatric nurses as not being genuine and authentic.

The personal change and empowerment of patients with mood disorders are vested in the therapeutic nurse–patient relationship. During group therapy, a therapeutic alliance is established between group members and the group facilitator. Group development can only evolve effectively if patients feel safe, respected and valued. Being friendly, approachable and portraying a skilled and experienced persona allows patients to be open and to express their most intimate feelings (Rørtveit et al. 2015:203).

Patients with mood disorders entered group therapy sessions feeling vulnerable, helpless and hopeless. Observing other group members’ emotional shift from being in distress to a stance of emotional well-being presented group members with a sense of hope and the possibility to experience emotional and physical well-being. Group therapy incorporating a vision of positive attributes in life, symptom relief and the potential to experience personal change and growth fosters a sense of hope (Hagen, Knizek & Hjelmeland 2017:33).

Patients struggling with mood disorders often isolate themselves; they feel alienated from others and their environment. In group therapy, participants had the opportunity to observe social cues from other group members. They also received feedback pertaining to their own social expressions and behavioural responses. According to Uys and Middleton (2015:243), the ability to interact functionally in an appropriate social context creates alliances of social support.

Humans have a deep-seated need to experience being valued and to be able to make valuable contributions to others and society. During group therapy, group members could experience making valuable contributions to the group therapy process itself and to the here-and-now experiences of other group members. Participants experienced enjoying time with others and became aware of the value of conducting pleasant activities.

### Patients’ experiences outside group therapy evolved around the perceived therapeutic environment and the interaction with psychiatric nurses and other group members outside the group setting

Literature refers to the therapeutic environment as the setting in which treatment is offered to patients, inclusive of the structural environment, patients and staff rendering the required service. In mental health care, the aim is to provide safe therapeutic care for patients with emotional distress and associated risks, for example self-destructive behaviour, suicidal tendencies and aggression directed at others (Shives 2008:153). In this study, the therapeutic environment refers to the inpatient unit for patients with mood disorders in a public psychiatric hospital in the Free State.

The environment in which patients with mood disorders are admitted should promote control, stabilisation and improvement in challenges related to emotional and behavioural responses with the aim to empower patients to effectively cope with intrapersonal and interpersonal challenges, trauma, losses and everyday life stressors. This study revealed that participants valued both the therapeutic space within the group settings as well as the therapeutic space outside the group setting. Participants clearly expressed that it was important for them to feel safe, supported and being valued even outside the group setting.

## Limitations of the study

Conducting this study in an acute admission unit for patients with mood disorders in a public psychiatric hospital posed specific challenges relating to the facilitation of group therapy by psychiatric nurses. Different patients were admitted and discharged at different times with the implication that some patients already attended a certain amount of sessions; therefore, newly admitted patients had to attend those sessions with another psychiatric nurse or at another time. Some participants were unwilling to participate in the study, especially those struggling with anxiety. The audio recording of the interviews seemed to aggravate their levels of anxiety.

## Recommendations

Patients admitted to acute inpatient units for mood disorders already experience emotional distress. It is therefore imperative for the psychiatric nurses conducting group therapy to minimise negative psychological experiences and enhance positive psychological experiences through an orientation process aimed at introducing patients to the benefits and principles underlying group therapy. Including activities of mastery and meaning into group therapy sessions will enhance empowerment and minimise feelings of helplessness, hopelessness and worthlessness.

Group therapy facilitated by psychiatric nurse should foster positive interpersonal experiences. Establishing sound therapeutic nurse–patient relationships within the group setting as well as outside the group setting has the ability to enhance trust, feeling safe enough to enter the therapeutic journey of therapeutic change offered by group therapy by psychiatric nurses.
